# The root-knot nematode effector MiPFN3 disrupts plant actin filaments and promotes parasitism

**DOI:** 10.1371/journal.ppat.1006947

**Published:** 2018-03-15

**Authors:** Natthanon Leelarasamee, Lei Zhang, Cynthia Gleason

**Affiliations:** 1 Department of Plant Molecular Biology and Physiology, Albrecht von Haller Institute, Georg August University, Göttingen, Germany; 2 Department of Plant Pathology, Washington State University, Pullman, WA, United States of America; University of California, Riverside, UNITED STATES

## Abstract

Root-knot nematodes secrete effectors that manipulate their host plant cells so that the nematode can successfully establish feeding sites and complete its lifecycle. The root-knot nematode feeding structures, their “giant cells,” undergo extensive cytoskeletal remodeling. Previous cytological studies have shown the cytoplasmic actin within the feeding sites looks diffuse. In an effort to study root-knot nematode effectors that are involved in giant cell organogenesis, we have identified a nematode effector called MiPFN3 (*Meloidogyne incognita* Profilin 3). *MiPFN3* is transcriptionally up-regulated in the juvenile stage of the nematode. In situ hybridization experiments showed that *MiPFN3* transcribed in the nematode subventral glands, where it can be secreted by the nematode stylet into the plant. Moreover, Arabidopsis plants that heterologously expressed *MiPFN3* were more susceptible to root-knot nematodes, indicating that *MiPFN3* promotes nematode parasitism. Since profilin proteins can bind and sequester actin monomers, we investigated the function of MiPFN3 in relation to actin. Our results show that MiPFN3 suppressed the aberrant plant growth phenotype caused by the misexpression of reproductive actin (*AtACT1*) in transgenic plants. In addition, it disrupted actin polymerization in an *in vitro* assay, and it reduced the filamentous actin network when expressed in Arabidopsis protoplasts. Over a decade ago, cytological studies showed that the cytoplasmic actin within nematode giant cells looked fragmented. Here we provide the first evidence that the nematode is secreting an effector that has significant, direct effects on the plant’s actin cytoskeleton.

## Author summary

Root-knot nematodes are microscopic plant pests that infect plant roots and significantly reduce yields of many crop plants. The nematodes enter the plant roots and modify plant cells into complex, multinuclear feeding sites called giant cells. The formation and maintenance of giant cells is critical to nematode survival. During giant cell organogenesis, the progenitor plant cells undergo many morphological changes, including a re-organization of the cytoplasmic actin cytoskeleton. As a result, the giant cell cytoplasmic actin appears fragmented and disorganized. Plant cells can regulate their actin filament assembly, in part, through the expression of actin binding proteins such as profilins. Here we show that infectious nematode juveniles express a profilin called MiPFN3. Expression of *MiPFN3* in Arabidopsis plants made the plants more susceptible to root-knot nematodes, indicating that MiPFN3 acts as an effector that aids parasitism. We show evidence that the expression *MiPFN3* in plant cells causes the fragmentation of plant actin filaments. The work here demonstrates that nematode effector MiPFN3 can directly affect plant actin filaments, whose reorganization is necessary for giant cell formation.

## Introduction

Root-knot nematodes (*Meloidogyne* spp) are small endoparasites with a host range that includes most flowering plants [1]. During the compatible interaction, the motile second stage juveniles (J2) enter the host roots and migrate intercellularly to the plant vasculature [2]. The primary nematode secretory organs, the esophageal glands, produce secretions. The gland secretions are exuded through the nematode stylet into the plant. Secretions that promote nematode parasitism are called effectors [3, 4]. Effectors help nematodes with successful parasitism by altering host defenses and/or modifying the plant cells to form the nematode feeding sites [3–5]. The feeding sites are comprised of 2–10 host cells (typically 6) that are reprogrammed to form large, multinucleate “giant cells” [6]. Root-knot nematodes are completely reliant on their giant cells as their sole source for food, and thus, the giant cells must be maintained throughout the nematode’s life for its survival.

As part of nematode effector research, several groups have worked to identify secreted root-knot nematode proteins and the plant processes that they affect [7–20]. Work from Bellafiore et al (2008) directly identified secreted proteins from *Meloidogyne incognita* by exposing the nematodes to root exudates before treating them with resorcinol to induce esophageal gland secretion [8]. Using sensitive mass spectrometry methods, 486 proteins were identified in the *M*. *incognita* “secretome” [8]. Although the proteins were categorized based on bioinformatic analyses, the roles of these potentially secreted proteins in plant-nematode interactions have been largely unexplored. Recently, Lin et al. (2016) used the secretome to identify a transthyretin-like protein 5 (TTL5). They showed that *M*. *javanica TTL5* homolog plays an important role in promoting parasitism by suppressing the host’s basal defense responses [21]. This work highlights the utility of studying the nematode secretome for identifying important nematode effectors.

Previous reports have used chemical and genetic experiments to show that actin cytoskeletal rearrangements are necessary for giant cell development [22, 23]. Therefore, we investigated it the nematodes were secreting effector(s) into the plant cells that were directly targeting actin filaments. In an effort to identify these effectors, we searched the published secretome for proteins that could interact with actin. Of the 486 peptides identified in the *M*. *incognita* secretome, 33 fell into the category “cell shape,” and predicted to interact with actin or microtubules [8]. Of these 33 proteins, two were annotated as profilins (PFNs): Proteins #131 and #240 [8].

Profilins are small actin binding proteins found in all eukaryotes whose main function is to bind globular (G) actin [24, 25]. Profilin can also bind to barbed ends of actin filaments, albeit with lower affinity than to G actin [26, 27]. In addition to binding to actin, profilin can also bind to polyphosphoinositide molecules, Arp2/3 complex, annexin, and proline-rich ligands [28–32]. Plant profilins do not share high amino acid similarity with profilins from other organisms, but they can complement profilin mutants in yeast and Dictyostelium, suggesting profilins have conserved functional roles across kingdoms [33, 34]. By focusing on nematode profilins found in the previously published secretome, we discovered that a nematode profilin, called MiPFN3, is an effector that facilitates parasitism. Our work shows that *MiPFN3* expression in plant cells causes a disruption to plant actin filaments.

## Results

### Identification of profilin genes in *M*. *incognita*

**Fig 1:**
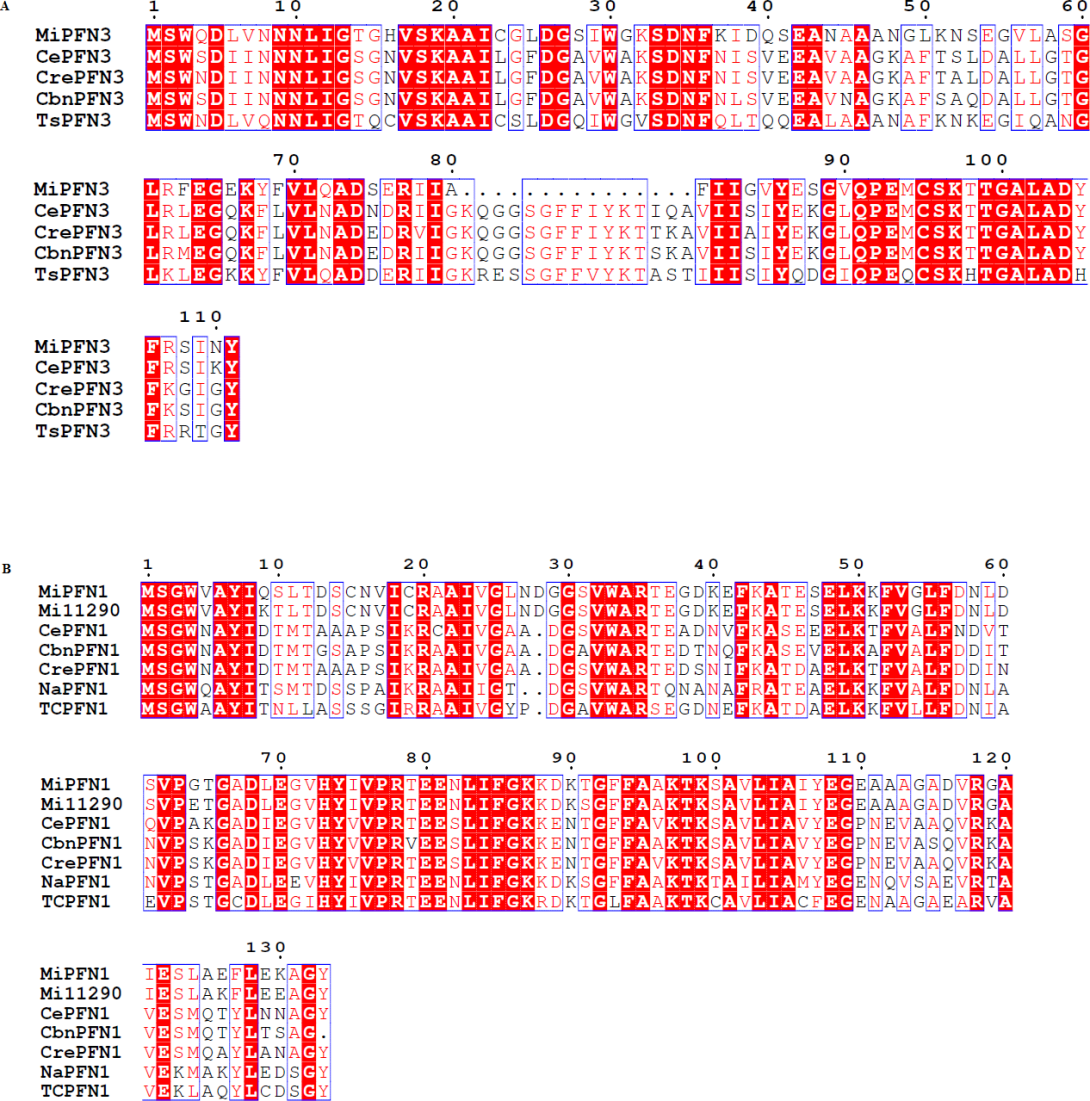
Multiple sequence alignment between two *M*. *incognita* profilins (MiPFN3 and MiPFN1) and their homologs in other nematodes. (A) Amino acid alignment of MiPFN3 with CePFN3, *C*. *elegans* PFN3 sequence K03E6.6; CrePFN3, *Caenorhabditis remanei* sequence CRE17080; CbnPFN3, *Caenorhabditis brenneri* sequence CBN01684; TsPFN3, *Trichinella spiralis* locus KRY72800. (B) Amino acid alignment of MiPFN1 with protein encoded by Minc11290, CePFN1, *C*. *elegans* sequence Y18D19.20; CrePFN1, *Caenorhabditis remanei* sequence CRE25828; CbnPFN1, *Caenorhabditis brenneri* sequence CBN24699; NaPFN1, *Necator americanus* sequence XP_013297957; TcPFN1, *Toxocara canis* sequence KHN75347. Strictly conserved residues are in white type on a red background; well-conserved residues are in red type, and conserved regions of residues are in blue boxes.

We were interested in specifically studying the two peptides found in the *M*. *incognita* secretome that were homologous *C*. *elegans* profilins (pfam00235): *M*. *incognita* #131 (Mi131) and #240 (Mi240) [8]. Using the peptide sequence for Mi131, a tBLASTn search of the Expressed Sequence Tag (est) database identified six ESTs with 100% identity to the Mi131 sequence (Genbank sequence IDs: JK291082.1, JK291081.1, JK267125.1, JK298994.1, JK303909.1, JK306024.1). These sequences contain an open reading frame of 381 bp that encodes a protein of 126 aa (S1 Fig). The protein sequence is 100% identical to the Mi131 peptide sequence, and it contains a profilin domain (pfam00235) (S1 Fig). When this protein sequence was used in a BLASTp search of *C*. *elegans* Sequencing Consortium genome project, the best hit was to *C*. *elegans* Profilin 3 (CePFN3), with 63.5% amino acid identity (Figs 1A and S2). In NCBI BLASTp search of Genbank’s non-redundant (nr) database with Mi131, the top four hits were to profilin 3 in parasitic and free-living nematodes: *Trichinella spiralis* PFN3, 65.6% identity; *C*. *elegans* PFN3, 63.5% aa identity; *Caenorhabditis remanei*, *-*PFN-3, 63.5% identity, and *Caenorhabditis brenneri* -PFN-3, 63.5% identity. Based on this sequence similarity to profilin 3 in *C*. *elegans* and other nematodes (Fig 1A), we refer to the gene as *MiPFN3*.

According to Bellafiore et al. (2008), Mi240 had a BLASTp top hit to *C*. *elegans* Profilin 1 (CePFN1). Using this information, we found significant tBLASTn hit in the *M*. *incognita* genome to one predicted full-length *M*. *incognita* gene, Minc11290, which encodes a 133 aa protein. Using primers based on this sequence, we amplified a 402 bp sequence. When this 402 bp sequence was used in a nucleotide blast search of the NCBI EST database, it was 100% identical to four *M*. *incognita* ESTs (Genbank Sequence IDs: JK295768.1, CF802658.1, CF802625.1, CK984306.1). This sequence encodes a 133 aa protein that is 95.5% identical to the protein encoded by Minc11290 (Fig 1B). In addition, a BLASTp search using the non-redundant (nr) database showed that the protein encoded by our amplified sequence had highest sequence homology to the hookworm profilin [*Necator americanus*, 70% aa identity]. There were also hits to *Toxocara canis* profilin [TC-PFN1 70% identity], *Caenorhabditis remanei* profilin, [CRE-PFN-1 protein 64% aa identity], and *Caenorhabditis brenneri* profilin [CBN-PFN-1 63% aa identity] (Figs 1B and S2). Based on this sequence homology to PFN1 in parasitic and free-living nematodes, we refer to the Mi240 protein as MiPFN1.

### MiPFN3 expression is up-regulated in second stage juveniles

**Fig 2:**
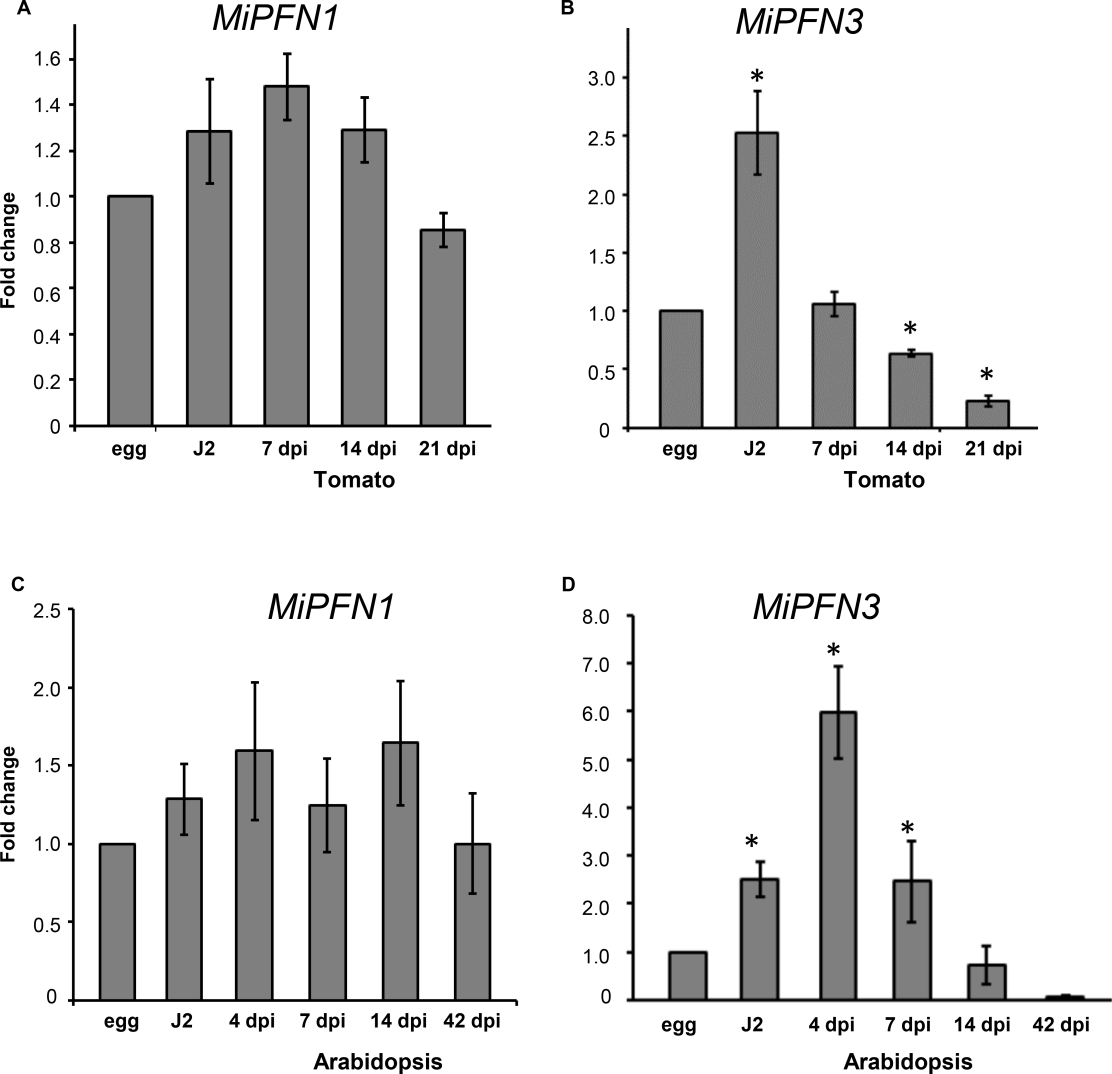
Expression of *M*. *incognita* profilin genes *MiPFN1* and *MiPFN3* in *M*. *incognita* at different life stages. The relative expression of (A) *MiPFN1* and (B) *MiPFN3* in eggs, J2, and nematodes inside tomato tissues at 7, 14, and 21 days post-inoculation was determined by Real-Time qRT-PCR. The relative expression of (C) *MiPFN1* and (D) *MiPFN3* in eggs, J2, and infective Arabidopsis roots at 4, 7, 14, and 42 dpi was also determined by Real-Time qRT-PCR. The fold change values were calculated using the 2-ΔΔCt method using reference gene *MiGAPDH*. Data presented as the change in expression relative to that of egg. The mean values (± SD) of the fold change for three biological replicates. *, *P*<0.05, One sample t-test.

Because genes involved in pathogenicity may be upregulated during the parasitic life stages of the nematode, we asked if either gene was up-regulated during nematode developmental life-stages associated with parasitism. Using primers specific for *MiPFN1* and *MiPFN3*, we measured the genes’ transcript levels in eggs, second stage juveniles (J2), and in nematodes-infected tomato roots at 7, 14, and 21 days post-inoculation, which represent the parasitic life-stages. The expression at the egg stage was set to 1 and used to calculate the fold change of the expression at the other time points. The expression of *MiPFN1* was not differentially expressed in the egg, J2 and in the parasitic nematodes life stages (Fig 2A). In contrast, *MiPFN3* expression was up-regulated in the J2 compared to the egg stage. In a later developmental life-stage (7 dpi), the *MiPFN3* expression level was equivalent to its expression in eggs (Fig 2B). Expression then decreased in nematodes in the 14 and 21 dpi samples.

To study earlier time points of nematode infection, we collected infected roots from Arabidopsis grown on MS-media at 4 dpi. We also collected infected roots at 7, 14, and 42 dpi. Acid fuchsin staining of the infected roots showed that at 4 dpi, the J2s penetrated the roots and were migrating as parasitic J2 (S3 Fig). At 7 dpi, visible galls had formed, but the parasitic nematodes still look like slim, non-feeding J2. By 14 dpi, we noticed that some nematodes had begun to looked fatter, an indication that the nematodes had initiated feeding. By 42 dpi, the nematode females had laid eggs in a gelatinous matrix on the surface of the root (S3 Fig). The expression of *MiPFN1* and *MiPFN3* was measured in the eggs, J2, and in the parasitic nematodes (4, 7, 14, 42 dpi). The expression at the egg stage was set to 1 and used to calculate the fold change of the expression at the other time points. Overall, *MiPFN1* was not differentially expressed at any time point (Fig 2C). However, *MiPFN3* expression was significantly up-regulated compared to the egg stage in the J2 and early parasitic stages (4 and 7 dpi) (Fig 2D). Because *MiPFN3* was strongly expressed in the J2, the infective stage of the nematode, and during early parasitic stages, it may be playing a role in the initial stages of plant parasitism, and therefore, we focused on *MiPFN3* for further characterization.

**Fig 3:**
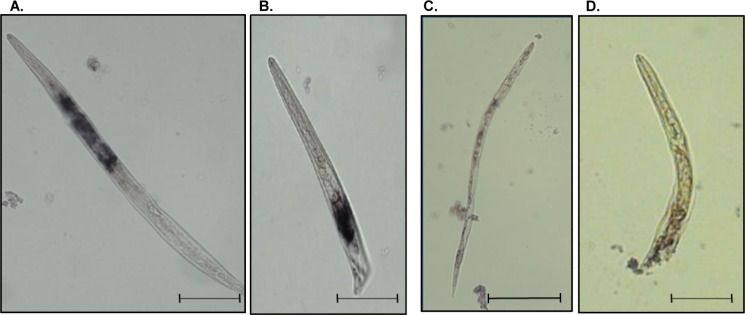
Subventral gland localization of MiPFN3 DIG-labeled cDNA probe in *M*. *incognita* J2. (A) and (B) representative photos of the MiPFN3 antisense probe binding to the subventral glands. Bar = 50 μm. (C) **and (D)** Sense probe of MiPFN3 did not hybridize to the nematode. Bar = 100 μm.

To determine where the *MiPFN3* transcript is expressed in the J2, we performed *in situ* hybridization using a DIG-labelled antisense *MiPFN3* cDNA probe on fixed juveniles. The probe hybridized to the esophageal glands (Fig 3A and 3B). The sense probe for *MiPFN3* did not hybridize to the nematodes (Fig 3C and 3D). Thus, we found *MiPFN3* transcripts present in a nematode secretory organ, which indicates that it encodes a protein secreted by *M*. *incognita*.

### Expression of *MiPFN3* in Arabidopsis makes the plants more susceptible to *M*. *incognita*

**Fig 4:**
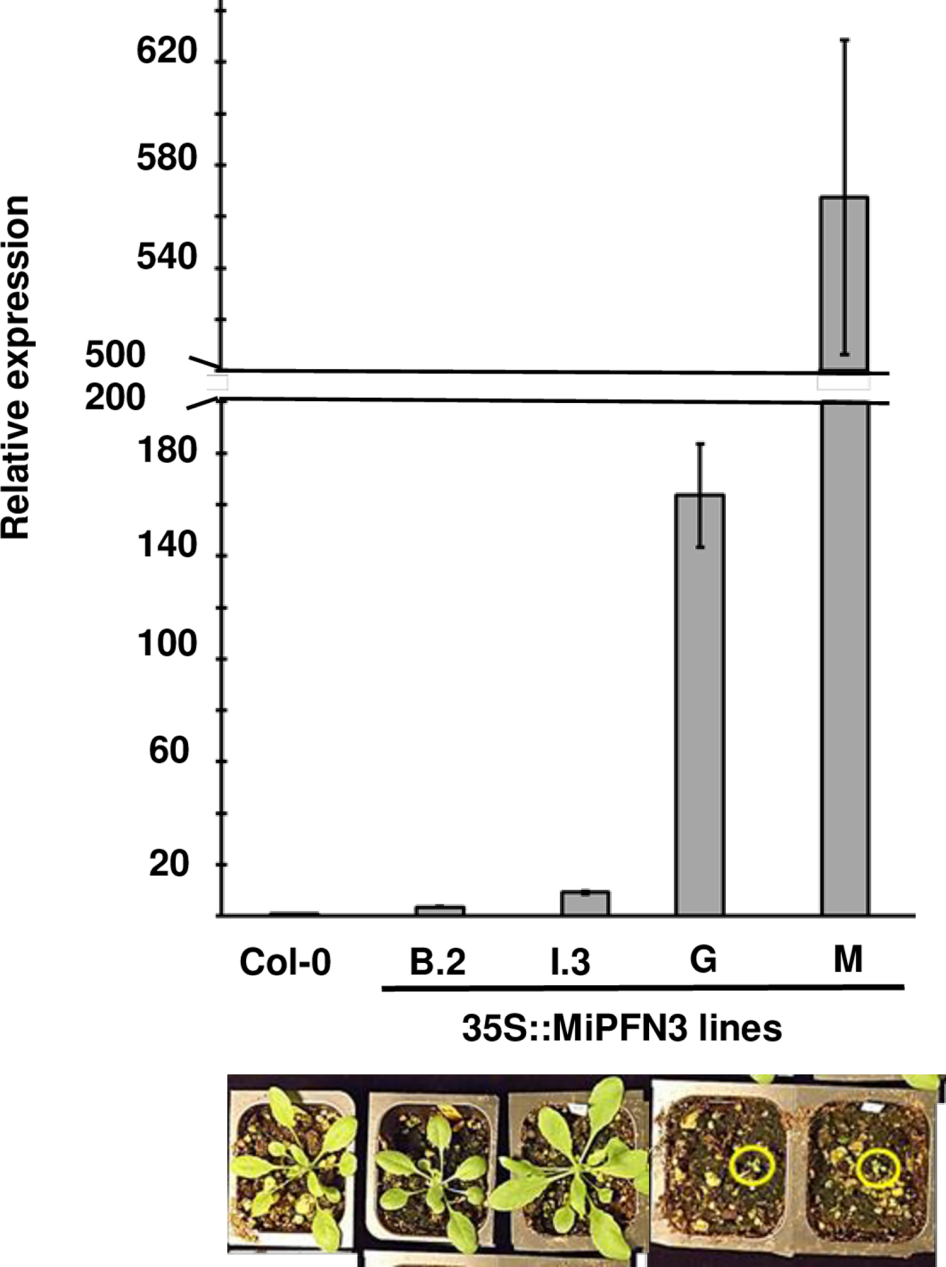
Correlation between *MiPFN3* expression in Arabidopsis and growth phenotype. (A) The relative expression of *MiPFN3* in Col-0 and the transgenic Arabidopsis lines B.2, I.3, G and M as determined by qRT-PCR. *AtUBQ5* was used as the reference gene. Bar represents mean of three biological replicates +/- sem. (B) Photos of 24 day old plants of Col-0 and transgenic Arabidopsis lines grown in soil. Circles indicate location of dwarf plants.

We next wanted to investigate if MiPFN3 has a role in nematode parasitism. Arabidopsis Col-0 was transformed with full-length *MiPFN3* driven by the Cauliflower Mosaic Virus 35S promoter. We obtained two T2 lines for plants with a dwarf phenotype, in which the rosettes were significantly smaller than Col-0 (lines G and M) (Fig 4). However, we were also able to generate two homozygous transgenic lines with single transgene insertion (MiPFN3 B.2 and I.3) and which did not exhibit size defects (Fig 4). The size of the plants corresponded to the amount of *MiPFN3* transcript measured in the plants, with lines G and M having significantly higher levels of *MiPFN3* than plants from lines B.2 and I.3, which had wild-type root growth (S4 Fig) and rosette sizes (Fig 4).

**Fig 5:**
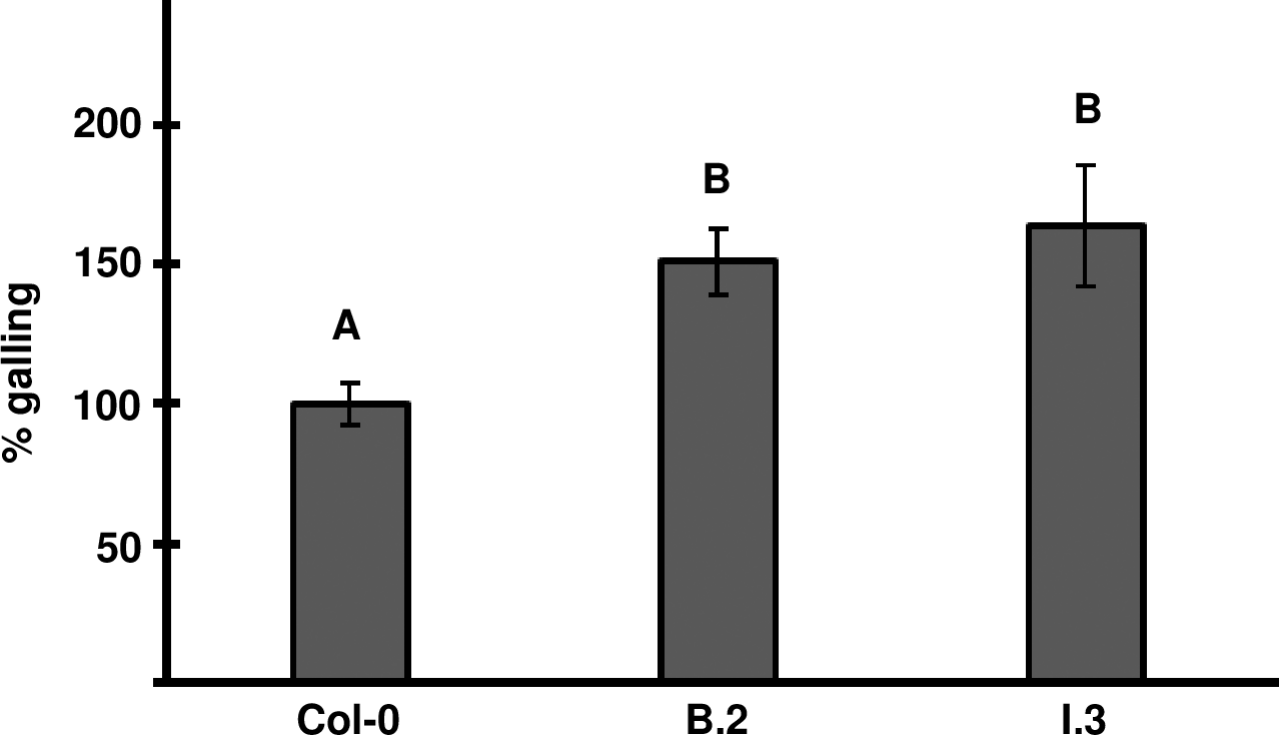
Transgenic Arabidopsis expressing *MiPFN3* are more susceptible to *M*. *incognita*. Two week old seedlings grown on MS media were inoculated with 100–150 *M*. *incognita* J2 and galling was quantified at 4 weeks post-infection. The relative number of galls in the transgenic lines B.2 and I.3 was higher than in the control Col-0. The average number of galls per plant in Col-0 was set 100%. Values are the means of at least 3 independent experiments +/- sem. (Col-0, n = 176; B-2, n = 161; I.3, n = 61). Different letters indicate significance using the Games-Howell Method and 95% Confidence.

Because the dwarf plants had smaller roots that can affect the number of nematode infection sites, we only tested lines that had wild-type root length phenotype in the root-knot nematode infection assays. Therefore, the T3 generation of MiPFN3 lines B.2 and I.3 were infected with *M*. *incognita*. Both independent transgenic lines showed increased levels of galling (Figs 5A and S4). The size of the galls in B.2 and I.3 were not significantly different to the control (Figs 5B and S5). There also was no obvious qualitative difference in the giant cells formed in the wild-type and transgenic lines (S6 Fig). Overall, the expression of MiPFN3 in the plant leads to increased number of galls, suggesting that MiPFN3 promotes nematode infections.

### MiPFN3 can suppress the AtACT1-mediated dwarf phenotype

**Fig 6:**
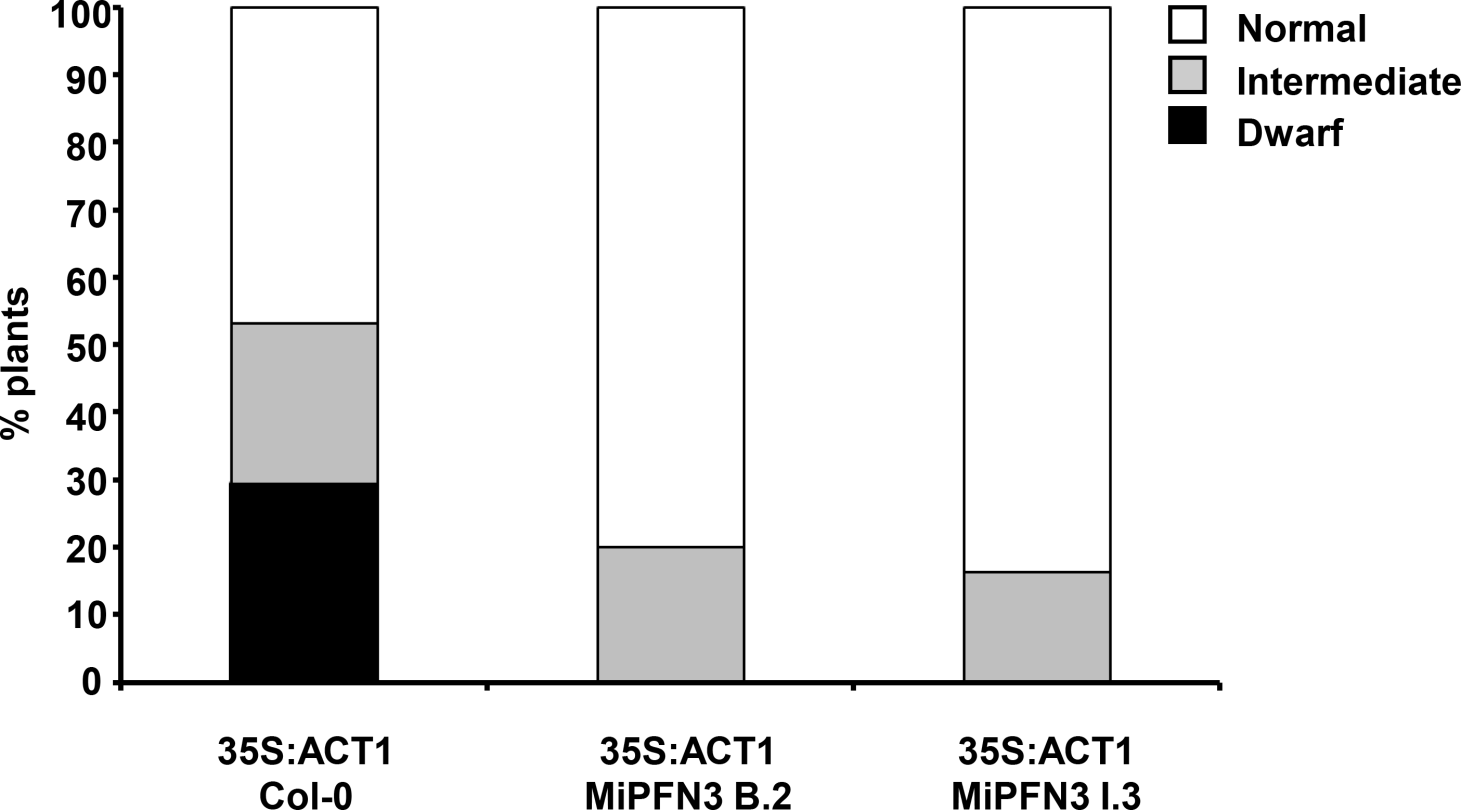
*MiPFN3* expression in plants suppresses the AtACT1-induced dwarf phenotype. Col-0 and transgenic MiPFN3 lines were transformed to express *35s*::*ACT1*. The growth phenotype of individual T1 plants was determined 5 weeks after germination. The number of dwarf, intermediate-sized, and wild-type rosette sizes were counted and normalized into a percentage of total plants (n = 109, 110 and 80 respectively).

A BLASTp search against the five profilin proteins in Arabidopsis showed that MiPFN3 had highest homology to Arabidopsis Profilin 4 AtPRF4 (38% amino acid identity) (S7 Fig). AtPRF4 can bind to actin monomers, and is specifically expressed in reproductive tissues (pollen and flowers) [35]. Based on its organ-specific expression pattern, AtPRF4 forms a complex with actin monomers that are also expressed in the plant’s reproductive organs (*ACT1*, *ACT3*, *ACT4*, *ACT11*and *ACT12*) [36]. A previous report showed that mis-expression of the reproductive actin ACT1 leads to an aberrant actin architecture in the plant, causing severe dwarfing of the plants. However, co-expression of AtPRF4 in these plants could suppress the ACT1-mediated dwarf phenotype [37]. Because MiPFN3 has similarity to AtPRFN4, we investigated whether MiPFN3 could also suppress the ACT1-induced dwarf phenotype. Arabidopsis Col-0 and two transgenic *MiPFN3* lines (B.2 and I.3) were transformed with *35S*::*AtACT1*. When the T1 seedlings started to produce inflorescences, the rosette size and the leaf morphology were graded into three categories: 1) small rosette (dwarf), 2) intermediate rosette size and 3) wild-type-like rosette size. When *AtACT1* was ectopically expressed in Col-0, approximately 30% of the T1 population exhibited a dwarf phenotype (Fig 6). When the transgenic *MiPFN3* lines were transformed with *35S*::*AtACT1*, none of the T1 plants exhibited a small rosette (Fig 6), indicating that MiPFN3 could suppress the AtACT1-induced dwarf phenotype.

### MiPFN3 affects the polymerization of soluble actin in an *in vitro* assay

**Fig 7:**
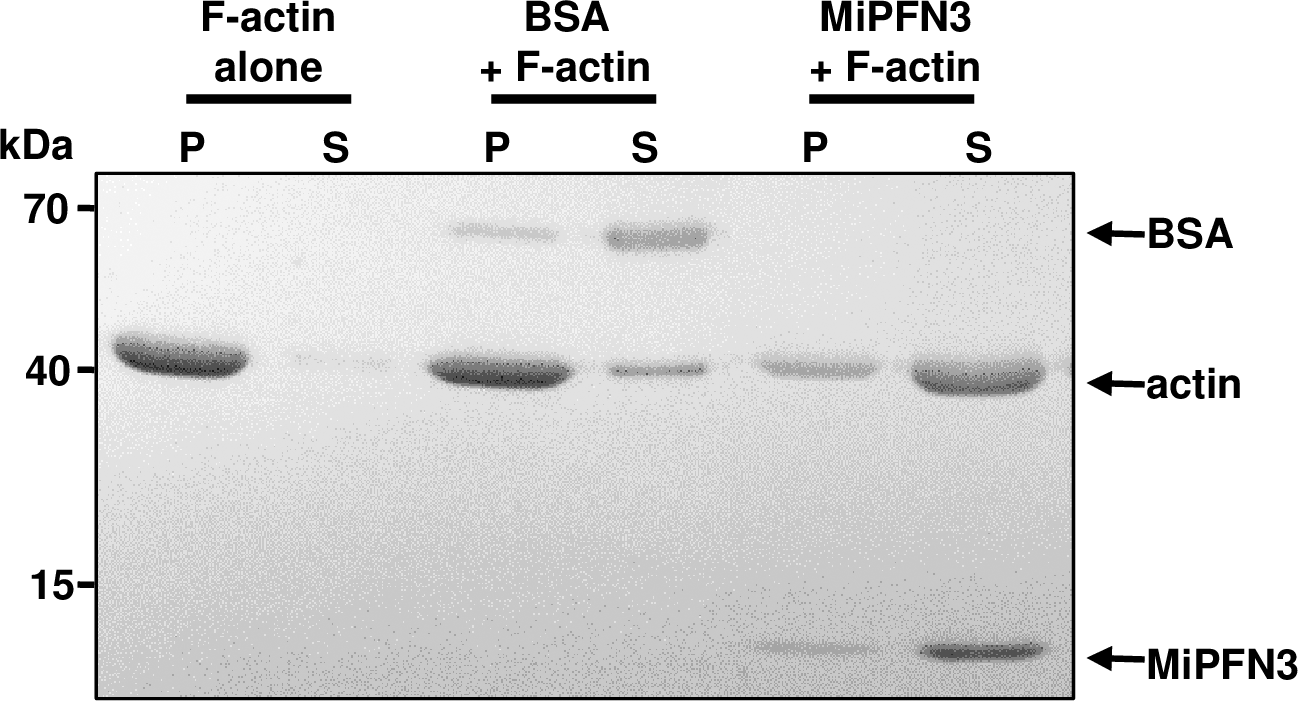
MiPFN3 affects actin polymerization *in vitro*. Non-muscular G actin (22μM) was incubated with BSA or 17 μM purified His-Mi131 for 30 minutes prior to actin polymerization. After actin polymerization, the samples were separated into the pellet (P) and supernatant (S) fractions by ultracentrifugation. The fractions were separated on a 4–20% SDS-PAGE and stained with Coomassie blue to visualize the proteins.

To clarify the effects of MiPFN3 on actin in more detail, *in vitro* actin sedimentation assays were performed using non-muscle actin and recombinant His-tagged MiPFN3. The His- MiPFN3 was added to soluble G actin prior to actin polymerization. After actin polymerization and sedimentation by centrifugation, we measured the ratio of soluble G actin to filamentous (F)-actin. When buffer or BSA (bovine serum albumin) were added to the G actin before polymerization, there was significantly more actin in the pellet fraction compared to the supernatant, indicating that most of the G actin had polymerized into F actin. On the other hand, when G actin was incubated prior to actin polymerization with purified recombinant MiPFN3, there was an increase in the G actin observed in the supernatant fraction after ultracentrifugation (Fig 7). In other words, relatively less F-actin polymerized if the actin monomers were incubated with MiPFN3 prior to polymerization.

### Ectopic expression of *MiPFN3* in plant cells disrupts the actin cytoskeleton

**Fig 8:**
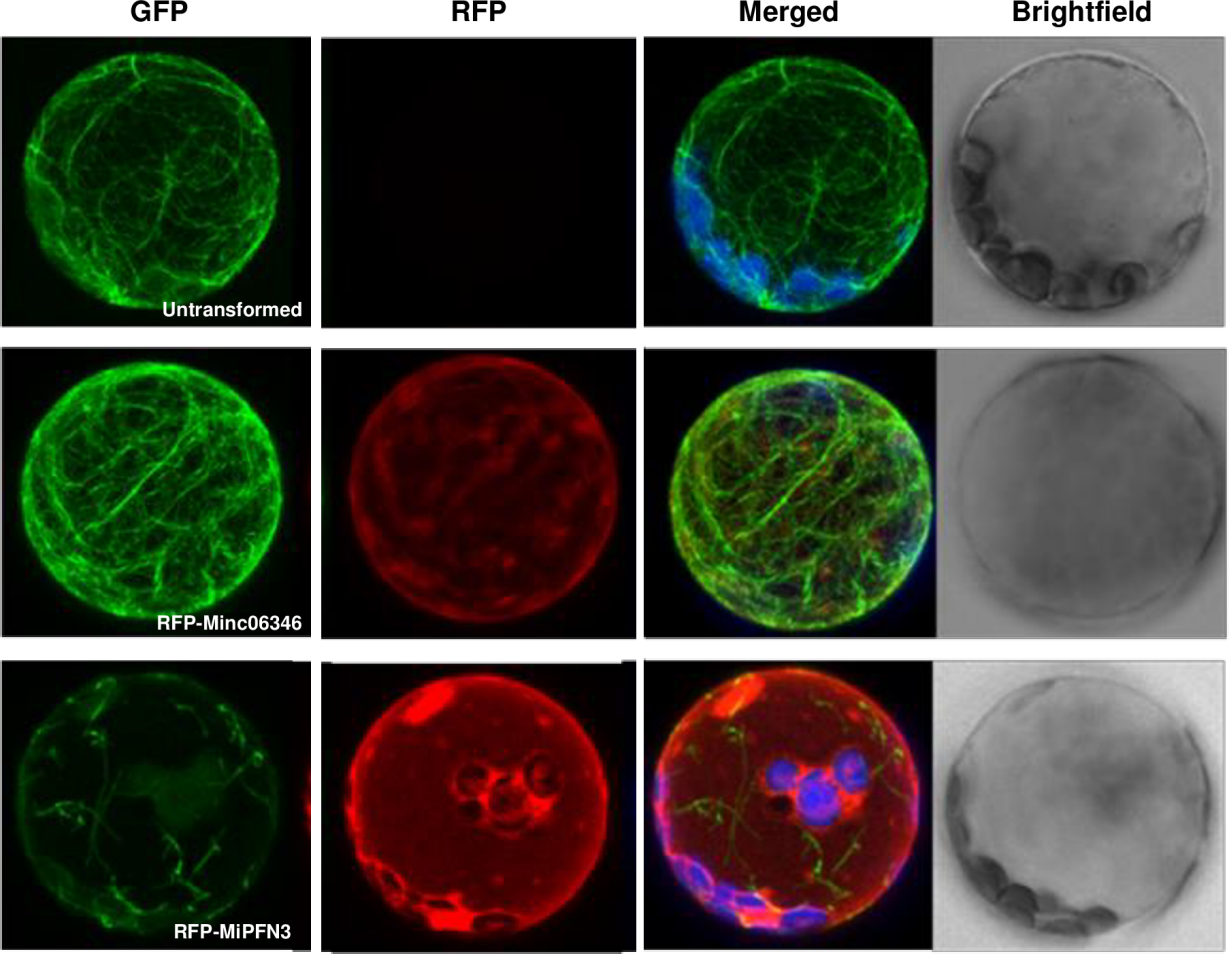
MiPFN3 disrupts the actin cytoskeleton in protoplasts. ABD2-GFP Arabidopsis protoplasts were transfected with RFP:Mi06346 or RFP:MiPFN3 and imaged by confocal microscopy (40x magnification). In the presence of MiPFN3, protoplasts showed altered actin filament structure. These experiments were performed at with at least 30 of ABD2-GFP protoplasts from each transfection and experiment was repeated at least twice times with similar results.

To study the effects *MiPFN3* expression on the actin cytoskeleton in plant cells, we expressed *MiPFN3* in Arabidopsis leaf protoplasts constitutively expressing *35S*::*ABD2-GFP*. The 35S::*ABD2-GFP* construct encodes a fibrin protein fused to GFP, and it can bind to and fluorescently label actin filaments [38]. We found that protoplasts expressing 35S::*RFP-MiPFN3* showed disrupted actin filaments and reduced the visible levels of ABD2-GFP compared to untransformed ABD2-GFP protoplasts, which showed dense, GFP-labeled F-actin (Fig 8).

To determine whether the effects on the actin cytoskeleton was specific for *MiPFN3* expression, the *M*. *incognita* Peptidyl-prolyl cis-trans isomerase (Minc06346) [39] was expressed in the ABD2-GFP protoplasts. The peptide corresponding to Minc06346 coding sequence was present in the *M*. *incognita* secretome [8], and the protein does not contain any predicted actin binding domains (Interpro Scan). When we transiently expressed full length cDNA Minc06346 driven by the 35S promoter fused at the N-terminus to RFP in the ABD2-GFP leaf protoplasts, the RFP fusion protein could be detected, and these cells had fluorescently labeled, dense actin filaments, similar to the un-transformed control (Fig 8). Therefore, *MiPFN3* expression in the protoplasts can specifically affect the organization and structure of the actin filaments.

## Discussion

We have identified a nematode profilin gene called *MiPFN3* as a novel nematode effector that is up-regulated in expression during parasitic life stages and when expressed in plants, it enhanced plant susceptibility to nematodes. MiPFN3 has homology to *C*. *elegans* profilin 3 (CePFN3) (Fig 1A). There are three profilin genes in the *C*. *elegans* genome. All three profilins are expressed in the worm and all behave as classical actin binding proteins [40]. In the *CePFN1* knockdown, cytokinesis of embryonic cells was affected [41], but the gene knockouts of *CePFN2* and *CePFN3* did not have phenotypes, indicating that these genes are non-essential [40]. The profilins in other free living and parasitic nematodes have not been characterized. Interestingly, the parasite *Toxoplasma gondii* possesses a profilin-like protein that is released by the parasite to facilitate its invasion of host cells [42, 43]. The profilin-like protein can bind actin, but it has also evolved a role at the host-parasite interface [42].

MiPFN3 was originally found in the root-knot nematode secretome [8]. Surprisingly, *MiPFN3* lacks a canonical secretion signal sequence. A root-knot nematode effector protein devoid of canonical secretion signal is not without precedent, and there are several examples of root-knot nematodes effectors, such as MI-14-3-3 and Mi-GSTS-1, which do not have canonical signal peptides but play key roles in plant-nematode interactions [20, 44–46]. The secretion of these proteins may be though a non-canonical secretory pathway that functions independently of the endoplasmic reticulum -Golgi network [20, 45, 46]. MiPFN3 may play a role in the secretome, and is expressed in the nematode esophageal glands, indicating that MiPFN3 is secreted from the nematode. Since these glands are connected by the esophagus to the stylet, it is possible for MiPFN3 to be delivered through the stylet directly into the plant [5].

In an effort to functionally characterize this profilin from root-knot nematodes, we performed in vitro actin polymerization assays. In these assays, actin-binding proteins bind and sequester actin monomers, preventing them from polymerizing into actin filaments. We found that pre-incubation of actin monomers with purified MiPFN3 prevented the formation of new actin filaments. Our results suggest that MiPFN3 sequesters G actin and inhibits nucleation of actin polymers in vitro.

Profilins are found in all eukaryotes, including plants [47]. Arabidopsis has five profilin genes that can be divided into two groups based on the tissues in which they are expressed: vegetative and reproductive [33, 37]. MiPFN3 has highest identity (38%) to AtPFN4 (At4g29340), a profilin expressed in plant reproductive organs. A previous report showed that the mis-expression of the reproductive actin *AtACT1* in plants caused dwarf plants, but overexpression of *AtPRF4* in these plants suppressed this phenotype [37]. By binding and sequestering the AtACT1 monomers, AtPRF4 prevented the deleterious effects on cytoskeletal architecture caused by reproductive actin misexpression [37, 48]. Because a root-knot nematode profilin that is secreted into the plant and may functionally mimic plant profilins, we tested MiPFN3 for its ability to suppress the ACT1-induced dwarf phenotype. Expression of *MiPFN3* in the 35S::*ACT1* plants resulted in plants with wild-type rosette size. Because MiPFN3 could suppress the dwarf morphological phenotype caused by ACT1 mis-expression, we conclude that MiPFN3 could bind to ACT1 *in planta* and sequester the excess reproductive actin monomers to a level that allowed for normal growth and development. When MiPFN3 was expressed at high levels in Col-0 (wild type), the resulting transgenic lines (G and M) showed growth defects (Fig 4). This indicates that MiPFN3 can also bind to the vegetative class of actin, which is found in vegetative organs such as leaves and roots. Overall, this data indicate MiPFN3 binds to both reproductive and vegetative classes of actin; there is no actin-class specific interaction between MiPFN3 and actin monomers.

We also wanted to investigate the effects of a nematode profilin on the actin filament dynamics of the plant. When *MiPFN3* was expressed in protoplasts, the GFP-labeled actin filaments appeared fragmented. In plant cells that were not transfected or transfected with another nematode gene, the actin cytoskeleton appeared intact. Thus, 35S::*MiPFN3* expression in Arabidopsis protoplasts affected the plant actin filaments. The fragmented actin phenotype was similar to the actin phenotype of *Tradescantia* stamen hair cells injected with birch profilin. The excess of birch profilin led to actin filament depolymerization and a reduction of actin microfilamants in the stamen hair cells [49, 50]. It was proposed that injected birch profilin sequestrated monomeric actin, leading to an inhibition actin polymerization and a depletion of F actin. Paradoxically, later studies showed that profilin facilitates actin polymerization by interacting with cytoskeletal proteins like formin and promoting the turnover of actin monomers. Profilin was also shown to promote actin filament assembly at the barbed-ends, competing with barbed end regulators and filament branching machinery [25, 27, 51, 52]. Thus, profilins regulate actin homeostasis through its roles in actin polymerization and depolymerization.

The effect on actin depends on the concentration of profilin and other actin binding proteins [53]. The strong expression of *MiPFN3* in the protoplasts may be disrupting the balance of profilin in the cell, and this leads to aberrant actin filaments. The transgenic lines with the highest level of *MiPFN3* expression showed aberrant, small rosettes, suggesting that the quantity of *MiPFN3* correlates with developmental defects [54, 55]. The two transgenic lines that had the lowest levels of *MiPFN3* transcript (B.2 and I.3) had roots that looked similar to wild type plants. Interestingly, although the lines B.2 and I.3 showed increased galling compared to the control, the overall gall phenotypes morphologies were not obviously different. Thus, *MiPFN3* has no role in gall expansion. Based on the expression of *MiPFN3* in the nematode during early parasitism, we postulate that *MiPFN3* facilitates early infection and feeding processes that lead to a higher percentage of nematodes that are successful in forming galls.

One possibility is that MiPFN3 plays a role in facilitating multinucleate giant cell formation and possibly maintenance. Previous cytological work showed that phragmoplasts, which act as scaffolding to support the newly formed cell wall between divided nuclei, are disordered and do not fully develop in giant cells [56–58]. The malformed phragmoplast results in aborted cell division and this leads to multinucleate giant cells [22, 23, 56, 59]. Work looking at the actin filaments associated with the phragmoplasts in giant cells showed that these actin filaments are disorganized [22]. Because the nematode effector MiPFN3 is linked to actin reorganization, MiPFN3 may be injected into the plant cell to play a role in the phragmoplast failure causing a blockage of cytokinesis in giant cells. Interestingly, cross sections of galls showed that giant cells in the wild-type and transgenic (B.2 and I.3) lines did not exhibit any obvious phenotypic differences (S6 Fig). The transgenic lines had relatively low levels of *MiPFN3* expression, and the lack of any obvious giant cell irregularities may reflect the delicate balance between the level of *MiPFN3* and actin monomers in the infected-transgenic plants. Our data showed that high levels of *MiPFN3* could tip the balance, leading to abnormal plant phenotypes. For example, high levels of *MiPFN3* in two transgenic lines (G and M) resulted in stunted growth, and strongly expressing *MiPFN3* in protoplasts affected the actin filaments. The *MiPFN3* in the transgenic lines B.2 and I.3 did not have a negative effect on galls size or giant cell phenotype, and *MiPFN3* may help nematodes to establish giant cells so that a higher percentage of infective juveniles are successful in infections and making galls.

In giant cells, the appearance of fragmented cytoplasmic actin filaments has been shown to be accompanied by transcriptional activation of actin and actin-related genes [22, 60–62]. Two representative of the actin gene family, *ACT2* and *ACT7*, are transcriptionally upregulated during giant cell development [22]. The up-regulation of these genes may be in response to wounding by nematode feeding or it may be suggestive that a pool of G-actin is necessary in feeding cells [22, 59]. In Arabidopsis, there is also an upregulation of formins *(AtFH1*, *AtFH6* and *AtFH10)*, which are involved in actin remodeling [56, 60, 61, 63]. The actin dynamics in the giant cells have also been linked with an increase in Arabidopsis actin-depolymerizing factor (ADF) gene expression. ADF/cofilins sever actin filaments and increase the rate at which actin monomers fall off the pointed end of the actin filaments [64]. Recently, specific ADF genes were also shown to be up-regulated in *M*. *incognita* infected cucumber (*Cucumis sativus* L) roots. The up-regulation of specific cucumber *ADF* genes correlated with the changes in plant actin structure that occurred during root-knot nematode infection [65]. Since ADFs can be involved in severing and depolymerizing actin filaments from their pointed ends, the increase ADF family gene expression in giant cells may be related to the fragmentation of the actin filaments that is observed in the feeding sites. In Arabidopsis, AtADF2 RNAi knockdowns exhibited an accumulation of actin bundles, and in these plants, feeding cell expansion was inhibited [60], indicating the important role of ADFs for nematode feeding site development. Considering the roles of ADFs and profilins, it may be possible that the nematode is enhancing the expression of endogenous ADFs to increase the pool of ADP–G-actin that can bind to MiPFN3.

This data suggest that diminishing the actin network density is important for facilitating nematode feeding. Up to now, the data has shown that the expression of plant genes, such those encoding formins and actin depolymerizing factors, can affect the organization of the actin filaments in giant cells. Because MiPFN3 is found in the secretome [8] and the transcript localizes to the esophageal glands, the protein is likely secreted into the plant. Here we have shown that a presumably secreted MiPFN3 can bind actin monomers to manipulate plant actin in conjunction with changes in plant gene expression.

## Materials and methods

### Nematode cultures

*Meloidogyne incognita (Morelos)* was used in all experiments. To collect nematode eggs, roots from infected tomato (*Solanum lycopersicum* Green Zebra) were mixed vigorously in 10% commercial bleach for 5 min. The eggs were collected on a 25 μm sieve and were further surface sterilized by vigorously shaking them in 10% bleach for 5 min. followed by three washes with sterile H_2_O. The bleach and wash steps were performed twice. After the last wash, the eggs were pelleted by a final centrifugation (4,000 rpm for 5 min) and re-suspended in 5 ml water with 0.1% SDS and 0.2% Plant Preservative Mixture (Plant Cell Technology). Freshly hatched J2 were collected on a modified Baermann Funnel as described [66].

### Transgenic lines

Sequences for *MiPFN1* and *MiPFN3* were obtained by BLAST searches of databases available online, such as WormBase, WormBase ParaSite [67] and NCBI, www.ncbi.nlm.nih.gov.

*M*. *incognita* J2 cDNA was the template for amplifying the coding sequences of MiPFN3 and the coding sequence for Minc06346 [39] by PCR. (See S1 Table for primer sequences). The amplified products were cloned into the Gateway pENTR Directional vector (Invitrogen) and then into the Gateway vectors pB2GW7, to generate constructs for Arabidopsis plant transformation, into the Gateway vector pB7WGR2, to generate the 35S::RFP-N terminal fusions for protoplast transformation [68], or into pDEST17 for expression in *Escherichia coli* for protein purification.

For stable plant transformation with 35S::MiPFN3, the construct was introduced to the *A*. *tumefaciens* strain GV3101 by heat shock transformation [69], and this was used to transform *Arabidopsis thaliana* Col-0 (N1093), using the floral dip method [70]. The seeds of the primary transformants were selected for BASTA resistance (Bayer CropScience, Wolfenbüttel, Germany). In the T2 generation, we selected lines segregating 3:1 (BASTA-resistant/BASTA-susceptible). At least seven BASTA resistant plants for each segregating T2 line were transferred to new pots. We found two lines that showed dwarf rosettes and two lines that had plants with normal growth and developmental phenotypes. The two wild-type looking lines were grown for seed, and homozygous lines were confirmed by 100% survival on BASTA-containing media in the T3 generation.

For the cloning of *AtACT1*, Arabidopsis Col-0 cDNA was used as the template for PCR, and the product was cloned into the entry vector pDONR207 and then destination vector pK2WG7. This construct was introduced to the *A*. *tumefaciens* strain GV3101 by heat shock transformation [69], which was then used to transform *Arabidopsis thaliana* Col-0 [70]. Seedlings (T1) from each background were first grown on plant media containing kanamycin to select for transformants containing the 35S::AtACT1 construct. At 10 days post germination on selective media, healthy plants were transferred to soil. When seedlings started to produce inflorescences (approx. 4 weeks at 14h light/10h dark, 22°C), the rosette size and the leaf morphology were graded into three categories: 1) severe abnormal leaf curling/small rosette, 2) intermediate rosette size and 3) wild-type-like rosette size.

### Nematode infections

Arabidopsis seeds were surface sterilized in 70% ethanol for 10 minutes, washed in 95% ethanol and allowed to air-dry. Seeds were placed on Murashige and Skoog media [71] with 2% sucrose and incubated in a growth chamber at 22°C/ 18°C, 80–100 μmol Photons/m^2^/s, 14h light/10h dark. The 14 day old seedlings were inoculated with 100–200 J2 of *M*. *incognita*. The inoculated plants were kept in the dark at 22°C as this facilitates infection for root-knot nematode bioassays [72]. Galls per root were counted at 4 weeks post-inoculation.

For sectioning, galls at 23 dpi were dissected from plants, fixed overnight at 4°C in 2% PFA, 2% GA 0.1M Cacodylate buffer. After the overnight incubation, fresh fixative was added to the samples and the samples were microwaved at 200 w until they reached 30°C. The samples were then incubated for 5 minutes at room temperature, rinsed then post-fixed with 1% OsO_4_ for overnight 4°C. The samples were dehydrated in an ethanol series (30% - 100%), then propylene oxide (PO). Galls were infiltrated with Spurrs resin prior to embedding and polymerization at 70°C overnight. Thick sections between 500 and 1000 nm were cut on a Leica EM UC7, stained for 45 seconds with 1% toluidine blue in 1% borax aq., then mounted for light microscopy. Samples were observed using a Zeiss Axio Observer A1 microscope.

### Quantitative Reverse-Transcriptase polymerase chain reaction (qRT-PCR)

Total RNA was extracted from eggs, freshly hatched J2, and gall enriched tissue. The gall enriched tissue was collected from infected tomato (Rutgers) roots grown in sand at greenshouse conditions at 7, 14, and 21 days post-inoculation (dpi). To monitor earlier time points, 2 week old Col-0 seedlings grown on MS were inoculated with freshly hatched nematodes. Root tissue was collected at 4, 7, 14, and 42 dpi. To monitor the nematode life stage in the Arabidopsis plants, the roots were stained with acid fuschin [73]. Nematodes and infected plant tissue from each time point was pooled for RNA extraction. cDNA synthesis and qRT-PCR was performed as previously described [74]. *MiPFN1* and *MiPFN3* expression was normalized to reference gene *MiGAPDH* [75]. Calculations were done according to the 2^–Δ*C*T^ method [76].

For qRT-PCR analysis of transgene expression in the stable transgenic lines, RNA extraction from Arabidopsis seedlings or whole plants (dwarves). qRT-PCR analysis for transgenic plants were performed as described [74, 77]. Calculations were done according to the 2^–Δ*C*T^ method. *AtUBQ5* served as a reference gene [78]. Primers for the qRT-PCR are listed in S1 Table.

### *In situ* hybridization

Using MiPFN3 purified PCR product as a template, an asymmetric PCR was performed in the presence of of DIG-labelled deoxynucleotide triphosphates (dNTPs) (Roche) to generate sense and anti-sense cDNA probes. In brief, the PCR contained 1x Advantage 2 buffer, 1x DIG-labelled nucleotides, 0.4 μM forward or reverse primer (The sense probe primer 5’-AACTGGCCATGTCTCAAAGG-3’; the anti sense probe primer 5’-TTAATAATTGATGCTTCGAAAGTAA-3’), approximately 200–800 ng PCR product, 1x Advantage 2 Polymerase Mix. The reaction performed for 1 cycle 95°C, 1 min and then 35 cycles at 95°C for 30 seconds, 59°C for 30 seconds, and 68°C for 30 seconds. The PCR probes were precipitated by mixing in 1 volume of 3M sodium acetate and 3 volumes 100% ethanol and kept at -20°C for at least one hour. The probe was centrifuged and the pellet resuspended in 300 μl hybridization buffer 50% deionized formamide, 4X SSC buffer, 1X Blocking Reagent, 2% SDS, 1X Denhardt's, 1 mm EDTA, pH 8, 200 μg/ml Fish sperm DNA, 3.125 yeast tRNA). Freshly hatched J2 were fixed and probed following the protocol of de Boer et al., 1988 [79]. The DIG-labeled probes were detected by incubation with the Alkaline phosphatase-conjugated anti-digoxigenin antibody (Roche Molecular Biochemicals) and the alkaline phosphatase substrate. Representative images were collected with a digital camera on a Leica microscope.

### Expression and purification of MiPFN3

The *E*. *coli* strain BL21 was transformed with either pDEST17-Mi131 (6xHis-Mi131). BL21 was cultured in 3 ml of LB + 100 μg/μl of ampicillin (Amp) overnight. The overnight culture was transferred into 30 ml of LB-Amp and grew until OD_600_ = 0.5. The protein expression was induced by adding 1 mM Isopropyl β-D-1-thiogalactopyranoside (IPTG) and incubating cells transformed with pDEST17-Mi131 construct at 37°C with 200 rpm shaking for 2 hours. Cells were harvested by centrifugation and resuspended in the lysis buffer and lysed by sonication at 60% power input for 5 minutes on ice. For the His-Mi131 purification, columns were prepared by adding 200 μl of Profinity IMAC resin into a Micro Bio-spin column. The spin columns were centrifuged at 1000g for 15 sec and washed with 250 μl deionized water. Columns were equilibrated by twice adding 250 μl of His purification wash buffer and centrifuging at 1000 x g for 15 sec. 200 μl of the bacterial lysate was added onto equilibrated columns and gently mixed by pipette. Lysates were incubated with resin for at least 5 minutes before centrifugation. The excess unbound proteins were removed by washing the column 3 more times with 250 μl of wash buffer. The bound protein was eluted with 100 μl of His purification elution buffer.

### Actin assays

Prior to the actin assays, the lysates, BSA, α-actin and purified His-Mi131 were prepared by ultracentrifugation at 150,000 x g for 60 min at 4°C and the supernatants were transferred into new Eppendorf tubes.

The G actin sequestration and F actin binding assays were performed following the manufacturer’s protocol (Cytoskeleton #BK013). In brief, a G actin solution was prepared by diluting 1 mg/ml of non-muscle actin with 225 μl of general actin buffer. The G actin solution was mixed by pipetting up and down several times and incubated on ice for 60 min prior to the assay. After the incubation, 40 μl of G actin solution was added into each tube with either 10 μl of test proteins or 10 μl of actin buffer. The mixture was mixed several times by pipetting up and down and incubated at RT for 30 mins. After the incubation, 2.5 μl of 10x polymerization buffer was added into each tube, mixed and incubated at room temperature for 30 min. To separate F actin from G actin, the mixtures were centrifuged at 150,000 x g for 90 min at 24°C. The supernatant was carefully removed and 5x reducing Laemmli buffer was added to each sample. The samples were centrifuged and the pellets were resuspended in 30 μl of Milli-Q water and incubated on ice for 10 min. Then 30 μl of 2 x Laemmli buffer was added to each sample. Samples were run on 4–20% SDS-gels and visualized by Coomassie staining.

### Protoplast assays

Arabidopsis Col-0 was transformed with pCAMBIA2300-ABD2 (Department of Cell Biology, Goettingen) using the floral dip method [70]. Approximately 10–15 leaves from 4–6 weeks old 35S::ABD2-GFP plant (T2), grown at 22°C/ 18°C, 80–100 μmol Photons/m^2^/s, 12h light/12h dark, 60% humidity, were collected. The leaf tissue was lysed using a 'Tape-*Arabidopsis* Sandwich' technique [80], in which pealed leaves were placed into 10 ml of enzyme solution (1.25% (w/v) Cellulase R-10, 0.3% Macerozyme R-10, 0.4 M mannitol, 20 mM KCl, 10 mM CaCl_2_, 20 mM MES (pH 5.7).The leaves were incubated at room temperature for 2 hours with constant slow rotation until the protoplasts were released into the enzyme solution. Then the protoplasts were carefully collected by centrifugation at 750 rpm for 5 minutes. The pellet was washed twice with 10 ml W5 buffer (2 mM MES (pH 5.7), 154 mM NaCl, 125 mM CaCl_2_, 5 mM KCl). The cells were chilled on ice for 30 minutes prior. Prior to PEG transformation, the W5 buffer (2 mM MES (pH 5.7), 154 mM NaCl, 125 mM CaCl_2_, 5 mM KCl) was removed by centrifugation, and the pellet was gently resuspended in 5 ml MMG buffer (4 mM MES (pH 5.7), 0.4 M mannitol, 15 mM MgCl_2_).

For PEG transfection of the protoplasts, up to 15.0 μg of the plasmid DNA was placed in a 2 ml Eppendorf tube containing 300 μl of 40% PEG 4000 solution and gently mixed with Protoplasts resuspended in 300 μl MMG buffer. The solution was gently mixed and incubated at 22°C for 30 minutes. At the end of the incubation, 800 μl of W5 buffer was added and gently mixed. The supernatant was removed after centrifugation at 750 rpm for 2 minutes and protoplasts were washed with 800 μl of WI buffer (4 mM MES (pH 5.7), 0.5 M mannitol, 20 mM KCl). The supernatant was removed and the pellet was suspended in 300-μl WI buffer, mixed gently and incubated at 22°C, overnight. On the next day, the incubated protoplasts were transferred onto a glass slide for the observation under the confocal laser scanning microscope (x40).

## Supporting information

S1 Fig MiPFN3 sequence information. (A) The nucleotide and amino acid sequence of *MiPFN3* cDNA. (B) Alignment of MiPFN3 amino acids with the profilin pfam00235. Identical residues between MiPFN3 and the profilin domain are in red font.
(PDF)

S2 Fig The percent identify matrix between *M*. *incognita MiPFN3*, *MiPFN1* and *C*. *elegans* protein sequences. There are three profilins in *C*. *elegans* (CePFN1, CePFN2, and CePFN3). *M*. *incognita* has MiPFN3, with highest homology to CePFN3 (64% aa identity). MiPFN1 has highest homology to CePFN1 (63.6%).
(PDF)

S3 Fig Nematode development inside Arabidopsis roots after infection at 4 dpi, 7 dpi, 14 dpi, and 42 dpi. Nematodes (arrows) and egg mass (em) are stained pink by acid fuchsin. Bar = 200 μm.
(PDF)

S4 Fig The MiPFN3 overexpressing lines B.2 and I.3 have normal root growth and are more susceptible to root-knot nematode infections. **(**A) Representative photo of 14-day-old seedlings Col-0 and lines B.2 and I.3. (B) The number of galls per plant at 14 dpi in Col-0 plants and the MiPFN3 transgenic lines B.2 and I.3. Values show the mean number of galls per plant ±SE for one representative experiment. n = 26 (Col-0), 11 (B.2) and 12 (I.3). * indicates a significant difference between Col-0 and the transgenic line using the Welch test (*p*<0.05).
(PDF)

S5 Fig The MiPFN3 transgenic lines B.2 and I.3 exhibit similar gall size (diameter) and qualitative giant cell structures as the wild-type Col-0. (A) Two week-old seedlings grown on MS media were inoculated with 125 *M*. *incognita* J2 / plant. The diameter of galls for wild-type and the two transgenic lines was measured at 23 dpi. Bar represents mean diameter (mm) ±SE. Means that share a letter are not significantly different, using Games-Howell Pairwise Comparisons. (B) Representative photos of galls from wild-type and transgenic lines B.2 and I.3 at 23 dpi. Scale bar = 0.5 mm.
(PDF)

S6 Fig Morphological analyses of giant cells at 23 dpi in *M*. *incognita*-infected Col-0, line B.2 and line I.3. Bright-field micrographs of tissue cross sections stained with toluidine blue. Bars = 20 μm. n, nematode; *, giant cell.
(PDF)

S7 Fig Sequence comparison between MiPFN3 and Arabidopsis profilins (AtPRF1-5). (A) Sequence alignment between MiPFN3 and AtPRF1 (At2g19760), AtPRF2 (At4g29350), AtPRF3 (At2g19760), AtPRF4 (At4g29340), and AtPRF5 (At2g19770). Alignment performed by ClustalOmega '*': Exact, ':': Conserved Substitution, '.': Semi-conserved substitution [81]. (B) Percent identity matrix for MiPFN3 and AtPRF1-5. MiPFN3 has highest amino acid identity to AtPRF4.
(PDF)

S1 Table Primers used in this study. (PDF)
