# Supplementary material for: The root-knot nematode effector MiPFN3 disrupts plant actin filaments and promotes parasitism
Source: PLoS Pathog. 2018 Mar 15;14(3):e1006947. doi: 10.1371/journal.ppat.1006947 (PMC5871015; doi:10.1371/journal.ppat.1006947)
Supplement: S1 Fig — (A) The nucleotide and amino acid sequence of MiPFN3 cDNA. (B) Alignment of MiPFN3 amino acids with the profilin pfam00235. Identical residues between MiPFN3 and the profilin domain are in red font. (PDF) [file ppat.1006947.s001.pdf]

A.

```
ATGTCTTGGCAAGATCTAGTTAACAACAACCTGATCGGAACTGGCCATGTCTCAAAGGCA
 M  S  W  Q  D  L  V  N  N  N  L  I  G  T  G  H  V  S  K  A

GCAATTTGTGGGCTCGATGGTTCTATTTGGGGGAAATCAGACAATTTTAAAATAGATCAA
 A  I  C  G  L  D  G  S  I  W  G  K  S  D  N  F  K  I  D  Q

TCGGAAGCAAATGCTGCAGCGAATGGACTTAAAAATTCAGAAGGTGTCCTAGCTTCCGGA
 S  E  A  N  A  A  A  N  G  L  K  N  S  E  G  V  L  A  S  G

TTGCGGTTTGAAGGCGAAAAATATTTTGTGCTACAAGCTGACTCTGAGCGGATAATAGGC
 L  R  F  E  G  E  K  Y  F  V  L  Q  A  D  S  E  R  I  I  G

AAAAAAACTGCCAATGGTTTCTTCATTTATAAAACGGACAAAGCATTTATAATTGGCGTT
 K  K  T  A  N  G  F  F  I  Y  K  T  D  K  A  F  I  I  G  V

TATGAGAGTGGAGTACAACCAGAAATGTGCAGTAAAACAACTGGCGCATTGGCTGATTAC
 Y  E  S  G  V  Q  P  E  M  C  S  K  T  T  G  A  L  A  D  Y

TTTCGAAGCATCAATTATTAA
 F  R  S  I  N  Y  *
```

B.

```
                      10        20        30        40        50        60        70        80
               ....*....|....*....|....*....|....*....|....*....|....*....|....*....|....*....|
MiPFN3     1  MSWQDLVNNNLIGTGHVSKAAICGLDG-SIWGKSDNFKIDQSEANAAANGLKNSEGVLASGLRFEGEKYFVLQADSERII 79
pfam00235  1  MSWQAYVDDNLVGTGHVDKAAIIGLDGgSVWAASPGFNLTPEEIKAIVAAFKDPSKLQANGITLGGEKYMVLRADDRSIY 80

                      90       100       110       120
               ....*....|....*....|....*....|....*....|....
MiPFN3     80 GKKTANGFFIYKTDKAFIIGVYESGVQPEMCSKTTGALADYFRS 123
pfam00235  81 GKKGKEGIVIVKTKQAIVIAHYDEGVQPGNANKAVEKLADYLRS 124
```

S1 Fig. *MiPFN3* sequence information. (A) The nucleotide and amino acid sequence of *MiPFN3* cDNA. (B) Alignment of MiPFN3 amino acids with the profilin pfam00235. Identical residues between MiPFN3 and the profilin domain are in red font.
