# Supplementary material for: The root-knot nematode effector MiPFN3 disrupts plant actin filaments and promotes parasitism
Source: PLoS Pathog. 2018 Mar 15;14(3):e1006947. doi: 10.1371/journal.ppat.1006947 (PMC5871015; doi:10.1371/journal.ppat.1006947)
Supplement: S2 Fig — There are three profilins in C. elegans (CePFN1, CePFN2, and CePFN3). M. incognita has MiPFN3, with highest homology to CePFN3 (64% aa identity). MiPFN1 has highest homology to CePFN1 (63.6%). (PDF) [file ppat.1006947.s002.pdf]

| | | | | | | |
|---|---|---|---|---|---|---|
| 1: MiPFN3 | 100 | 64.00 | 22.31 | 29.84 | 26.83 | 29.03 |
| 2: CePFN3 | 64.00 | 100 | 27.05 | 32.80 | 27.42 | 33.60 |
| 3: CePFN2 | 29.84 | 32.80 | 38.58 | 100 | 57.69 | 57.25 |
| 4: CePFN1 | 26.83 | 27.42 | 41.27 | 57.69 | 100 | 63.64 |
| 5: MiPFN1 | 29.03 | 33.60 | 57.48 | 57.25 | 63.64 | 100 |

**S2 Fig. The percent identify matrix between *M. incognita MiPFN3, MiPFN1* and *C. elegans* protein sequences.** There are three profilins in *C. elegans* (CePFN1, CePFN2, and CePFN3). *M. incognita* has MiPFN3, with highest homology to CePFN3 (64% aa identity). MiPFN1 has highest homology to CePFN1 (63.6%).
