# Supplementary material for: The root-knot nematode effector MiPFN3 disrupts plant actin filaments and promotes parasitism
Source: PLoS Pathog. 2018 Mar 15;14(3):e1006947. doi: 10.1371/journal.ppat.1006947 (PMC5871015; doi:10.1371/journal.ppat.1006947)
Supplement: S3 Fig — Nematodes (arrows) and egg mass (em) are stained pink by acid fuchsin. Bar = 200 μm. (PDF) [file ppat.1006947.s003.pdf]

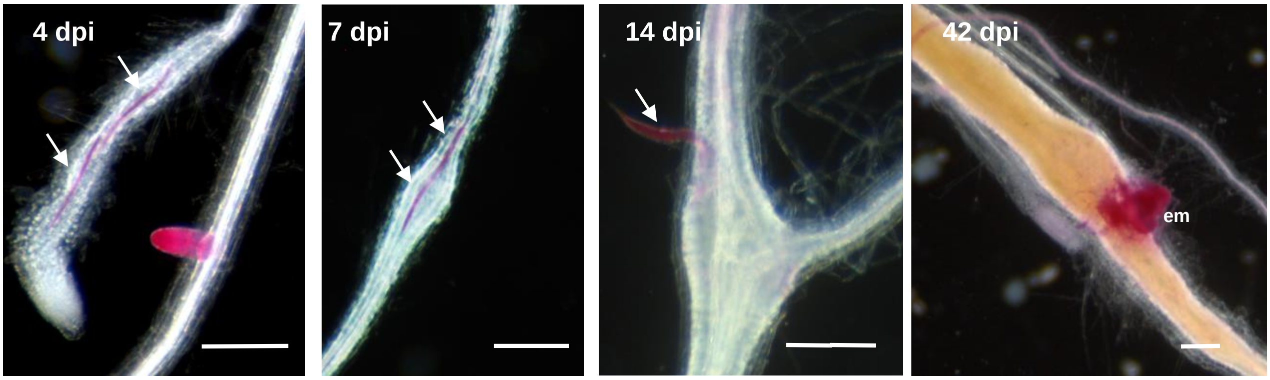

S3 Fig. Nematode development inside Arabidopsis roots after infection at 4 dpi, 7 dpi, 14 dpi, and 42 dpi. Nematodes (arrows) and egg mass (em) are stained pink by acid fuchsin. Bar = 200 µm
