# Supplementary material for: The root-knot nematode effector MiPFN3 disrupts plant actin filaments and promotes parasitism
Source: PLoS Pathog. 2018 Mar 15;14(3):e1006947. doi: 10.1371/journal.ppat.1006947 (PMC5871015; doi:10.1371/journal.ppat.1006947)
Supplement: S4 Fig — (A) Representative photo of 14-day-old seedlings Col-0 and lines B.2 and I.3. (B) The number of galls per plant at 14 dpi in Col-0 plants and the MiPFN3 transgenic lines B.2 and I.3. Values show the mean number of galls per plant ±SE for one representative experiment. n = 26 (Col-0), 11 (B.2) and 12 (I.3). * indicates a significant difference between Col-0 and the transgenic line using the Welch test (p<0.05). (PDF) [file ppat.1006947.s004.pdf]

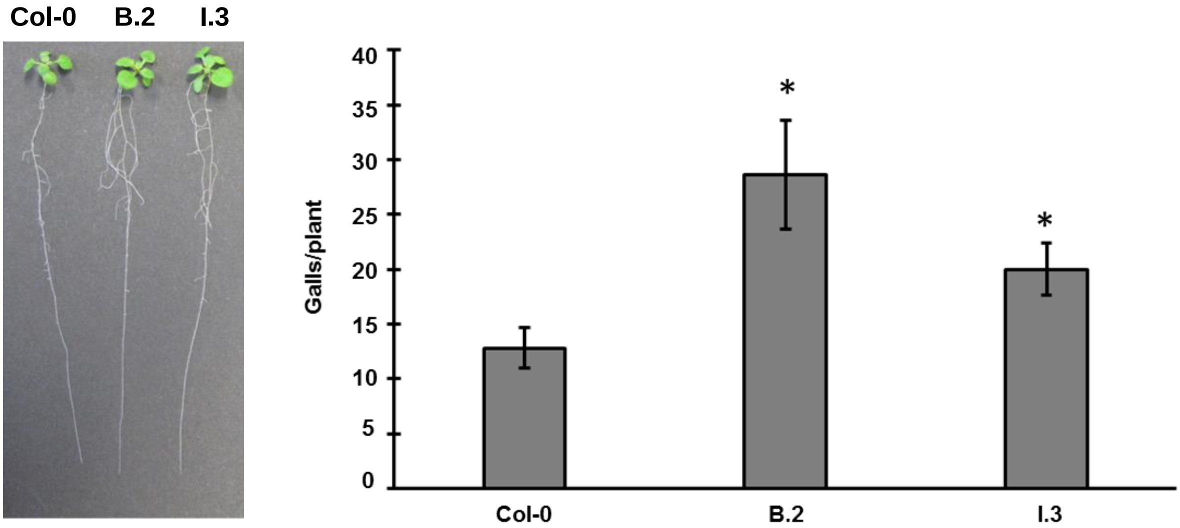

S4 Fig. The *MiPFN3* overexpressing lines B.2 and I.3 have normal root growth and are more susceptible to root-knot nematode infections. (A) Representative photo of 14 day old seedlings Col-0 and lines B.2 and I.3. (B) The number of galls per plant at 14 dpi in Col-0 plants and the MiPFN3 transgenic lines B.2 and I.3. Values show the mean number of galls per plant ±SE for one representative experiment. n=26 (Col-0), 11 (B.2) and 12 (I.3). * indicates a significant difference between Col-0 and the transgenic line using the Welch test (p<0.05).
