# Supplementary material for: The root-knot nematode effector MiPFN3 disrupts plant actin filaments and promotes parasitism
Source: PLoS Pathog. 2018 Mar 15;14(3):e1006947. doi: 10.1371/journal.ppat.1006947 (PMC5871015; doi:10.1371/journal.ppat.1006947)
Supplement: S5 Fig — (A) Two week-old seedlings grown on MS media were inoculated with 125 M. incognita J2 / plant. The diameter of galls for wild-type and the two transgenic lines was measured at 23 dpi. Bar represents mean diameter (mm) ±SE. Means that share a letter are not significantly different, using Games-Howell Pairwise Comparisons. (B) Representative photos of galls from wild-type and transgenic lines B.2 and I.3 at 23 dpi. Scale bar = 0.5 mm. (PDF) [file ppat.1006947.s005.pdf]

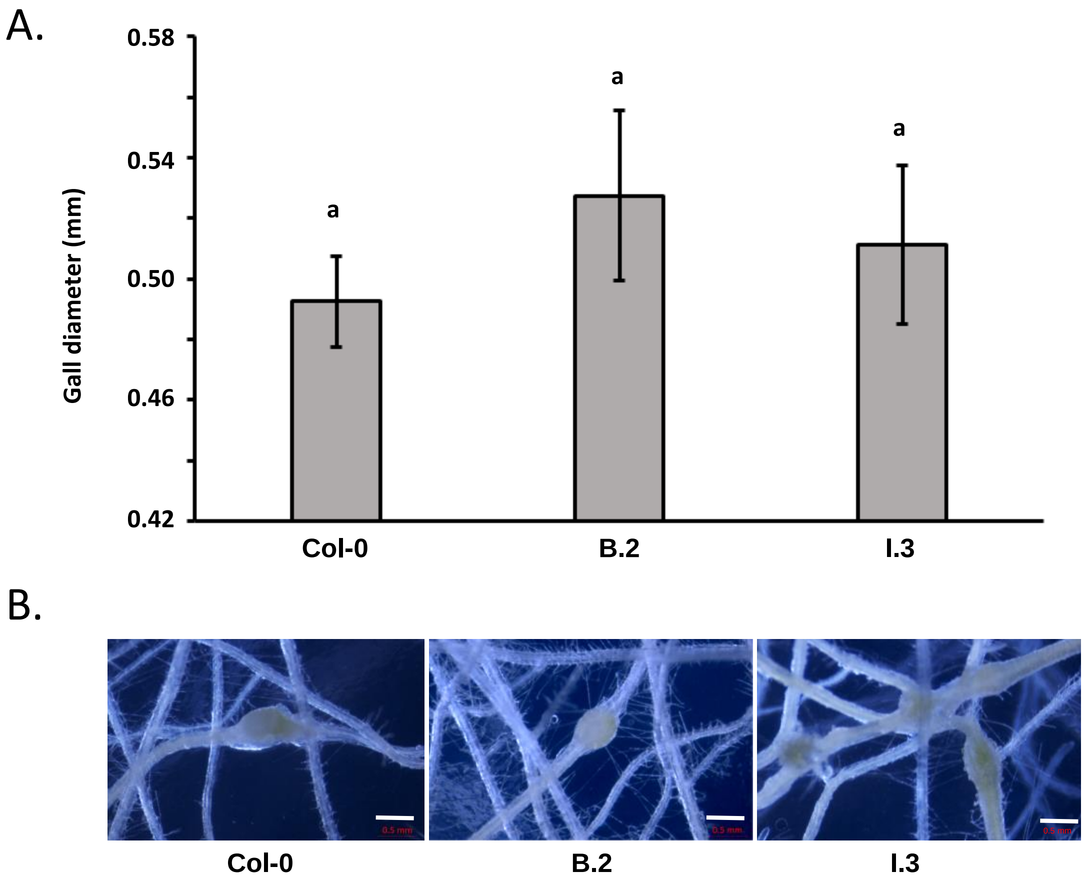

S5 Fig. The *MiPFN3* transgenic lines B.2 and I.3 exhibit similar gall size (diameter) and qualitative giant cell structures as the wild-type Col-0. (A) Two week-old seedlings grown on MS media were inoculated with 125 *M. incognita* J2 / plant. The diameter of galls for wild-type and the two transgenic lines was measured at 23 dpi. Bar represents mean diameter (mm) ±SE. Means that share a letter are not significantly different, using Games-Howell Pairwise Comparisons. (B) Representative photos of galls from wild-type and transgenic lines B.2 and I.3 at 23 dpi. Scale bar = 0.5 mm.
