# Supplementary material for: The root-knot nematode effector MiPFN3 disrupts plant actin filaments and promotes parasitism
Source: PLoS Pathog. 2018 Mar 15;14(3):e1006947. doi: 10.1371/journal.ppat.1006947 (PMC5871015; doi:10.1371/journal.ppat.1006947)
Supplement: S6 Fig — Bright-field micrographs of tissue cross sections stained with toluidine blue. Bars = 20 μm. n, nematode; *, giant cell. (PDF) [file ppat.1006947.s006.pdf]

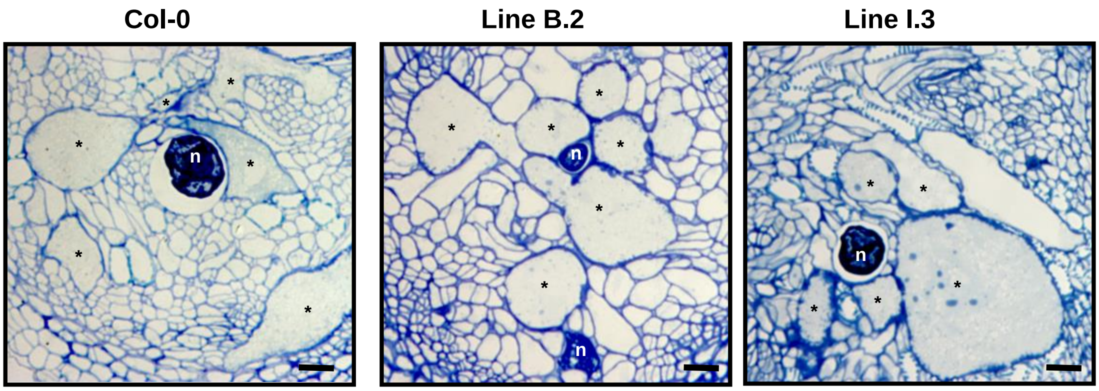

S6 Fig. Morphological analyses of giant cells in infected (23 dpi) Col-0, Line B.2 and Line I.3. Bright-field micrographs of tissue cross sections stained with toluidine blue. Bars = 20 µm. n, nematode; *, giant cell.
