# Supplementary material for: The root-knot nematode effector MiPFN3 disrupts plant actin filaments and promotes parasitism
Source: PLoS Pathog. 2018 Mar 15;14(3):e1006947. doi: 10.1371/journal.ppat.1006947 (PMC5871015; doi:10.1371/journal.ppat.1006947)
Supplement: S7 Fig — (A) Sequence alignment between MiPFN3 and AtPRF1 (At2g19760), AtPRF2 (At4g29350), AtPRF3 (At2g19760), AtPRF4 (At4g29340), and AtPRF5 (At2g19770). Alignment performed by ClustalOmega '*': Exact, ':': Conserved Substitution, '.': Semi-conserved substitution [81]. (B) Percent identity matrix for MiPFN3 and AtPRF1-5. MiPFN3 has highest amino acid identity to AtPRF4. (PDF) [file ppat.1006947.s007.pdf]

A.

```
MiPFN3       MSWQDLVNNNLIG------TGHVSKAAICGLDGSIWGKSDNFKIDQSEAN-AAANGLKNS
AtPRF4       MSWQTYVDEHLMCDVGDGQGHHLTAAAIVGHDGSVWAQSANFPQFKGQEFSDIMKDFDEP
AtPRF5       MSWQAYVDEHLMCDVGDGQGHHLTAAAIIGHDGSVWAQSANFPQFKPQEITDIMKDFDEP
AtPRF3       MSWQTYVDDHLMCDVA---GNRLTAAAILGQDGSVWAQSNNFPQVKPEEIQGIKDDFTTP
AtPRF2       MSWQSYVDDHLMCEVE---GNHLTHAAIFGQDGSVWAQSSAFPQLKPAEIAGINKDFEEA
AtPRF1       MSWQSYVDDHLMCDVE---GNHLTAAAILGQDGSVWAQSAKFPQLKPQEIDGIKKDFEEP
             ****  *:::*:         :::  *** * ***:*.:*  *   :        ..:

MiPFN3       EGVLASGLRFEGEKYFVLQADSER-IIGKKTANGFFIYKTDKAFIIGVYESGVQPEMCSK
AtPRF4       GHLAPTGLFMAGAKYMVIQGEPGAVIRGKKGAGGITIKKTGQSCVFGIYEEPVTPGQCNM
AtPRF5       GHLAPTGMFLAGLKYMVIQGEPNAVIRGKKGAGGITIKKTGQSMVFGLYEEPVTPGQCNM
AtPRF3       GTLAPTGLFLGGNKYMVIQGEPNAVIRGKKGAGGVTIKKTTLALVFGIYDEPMTPGQCNM
AtPRF2       GHLAPTGLFLGGEKYMVVQGEAGAVIRGKKGPGGVTIKKTTQALVFGIYDEPMTGGQCNL
AtPRF1       GFLAPTGLFLGGEKYMVIQGEQGAVIRGKKGPGGVTIKKTNQALVFGFYDEPMTGGQCNL
               :  :*: : * **:*:*.:    * ***  .*. * **  : ::*.*:. :    *.

MiPFN3       TTGALADYFRSINY
AtPRF4       VVERLGDYLLEQGL
AtPRF5       VVERLGDYLIEQGL
AtPRF3       VVENLGEYLIESGL
AtPRF2       VVERLGDYLIESGL
AtPRF1       VVERLGDYLIESEL
             ..  *.:*: .
```

B.

1: MiPFN3	100	34.13	33.33	32.54	31.75	31.75
2: AtPRF4	34.13	100	91.04	74.81	73.28	75.57
3: AtPRF5	33.33	91.04	100	76.34	74.05	77.10
4: AtPRF3	32.54	74.81	76.34	100	79.39	81.68
5: AtPRF2	31.75	73.28	74.05	79.39	100	88.55
6: AtPRF1	31.75	75.57	77.10	81.68	88.55	100

S7 Fig. Sequence comparison between MiPFN3 and Arabidopsis profilins (AtPRF1-5). (A) Sequence alignment between MiPFN3 and AtPRF1 (At2g19760), AtPRF2 (At4g29350), AtPRF3 (At2g19760), AtPRF4 (At4g29340), and AtPRF5 (At2g19770). Alignment performed by ClustalOmega '*': Exact, ':': Conserved Substitution, '.': Semi-conserved substitution. (Goujon, M et al., 2010). (B) Percent identity matrix for MiPFN3 and AtPRF1-5. MiPFN3 has highest amino acid identity to AtPRF4.
