# Supplementary material for: The root-knot nematode effector MiPFN3 disrupts plant actin filaments and promotes parasitism
Source: PLoS Pathog. 2018 Mar 15;14(3):e1006947. doi: 10.1371/journal.ppat.1006947 (PMC5871015; doi:10.1371/journal.ppat.1006947)
Supplement: S1 Table — (PDF) [file ppat.1006947.s008.pdf]

S1 Table. Primers used in this study.

Primer	Forward sequence 5'-3'	Reverse sequence 5'-3'
1. MiPFN3 coding sequence	CACCATGTTTCGAAATCAGTATGATAGTG	TTAATAATTGATGCTTCGAAAGTAA
2. MiPFN3 qRT-PCR	TGGGCTCGATGGTTCTATTT	ATCCGGAAGCTAGGACACCT
3. MiPFN1 qRT-PCR	CGAGTCGGAACTCAAGAAGT	TGCCAAAAATGAGGTTCTCC
4. Minc06346 coding sequence	GGGGACAAGTTTGTACAAAAAAGCAGGCTCCATGCCAGTCCAAATTGATACTATAAATG	GGGGACCACTTTGTACAAGAAAGCTGGGTCTTATTTGACAGCAAGCAATTCAAC
5. GAPDH qRT-PCR	TCTACCCACGGACGTTTCAA	CGGAGCTGAGATAACCACCT
6. MiPFN3-in situ sense	AACTGGCCATGTCTCAAAGG	
7. MiPFN3 in situ antisense probe		TTAATAATTGATGCTTCGAAAGTAA
8. Arabidopsis UBQ5	GACGCTTCATCTCGTCC	GTAAACGTAGGTGAGTCCA
9. ACT1 gateway primer	GGGGACAAGTTTGTACAAAAAAGCAGGCTTCAACATGGCTGATGGTGAAGACATTC	GGGGACCACTTTGTACAAGAAAGCTGGGTCTCAGAAGCACTTCCTGTGAACA
